# Evaluation of bacteriocinogenic activity, safety traits and biotechnological potential of fecal lactic acid bacteria (LAB), isolated from Griffon Vultures (*Gyps fulvus* subsp. *fulvus*)

**DOI:** 10.1186/s12866-016-0840-2

**Published:** 2016-09-29

**Authors:** Sara Arbulu, Juan J. Jiménez, Loreto Gútiez, Cristina Campanero, Rosa del Campo, Luis M. Cintas, Carmen Herranz, Pablo E. Hernández

**Affiliations:** 1 Departamento de Nutrición, Bromatología y Tecnología de los Alimentos, Facultad de Veterinaria, Universidad Complutense de Madrid (UCM), Avenida Puerta de Hierro, s/n, 28040 Madrid, Spain; 2 Servicio de Microbiología, Hospital Universitario Ramón y Cajal, and Instituto Ramón y Cajal de Investigaciones Sanitarias (IRYCIS), 28034 Madrid, Spain

**Keywords:** Lactic acid bacteria (LAB), Antimicrobial activity, Bacteriocins, Enterococci, Virulence traits, Probiotics

## Abstract

**Background:**

Lactic acid bacteria (LAB) are part of the gut microbiota and produce ribosomally synthesized antimicrobial peptides or bacteriocins with interest as natural food preservatives and therapeutic agents. Bacteriocin-producing LAB are also attractive as probiotics. Griffon vultures (*Gyps fulvus* subspecies *fulvus*) are scavenger birds that feed almost exclusively on carrion without suffering apparent ill effects. Therefore, griffon vultures might be considered a reservoir of bacteriocin-producing lactic acid bacteria (LAB) with potential biotechnological applications.

**Results:**

Griffon vulture feces were screened for LAB with antimicrobial activity, genes encoding bacteriocins, potential virulence determinants, susceptibility to antibiotics, genotyping and characterization of bacteriocins. In this study, from 924 LAB evaluated 332 isolates (36 %) showed direct antimicrobial activity against Gram-positive bacteria only. The molecular identification of the most antagonistic 95 isolates showed that enterococci was the largest LAB group with antimicrobial activity (91 %) and *E. faecium* (40 %) the most identified antagonistic species. The evaluation of the presence of bacteriocin structural genes in 28 LAB isolates with the highest bacteriocinogenic activity in their supernatants determined that most enterococcal isolates (75 %) encoded multiple bacteriocins, being enterocin A (EntA) the largest identified (46 %) bacteriocin. Most enterococci (88 %) were resistant to multiple antibiotics. ERIC-PCR and MLST techniques permitted genotyping and recognition of the potential safety of the bacteriocinogenic enterococci. A multiple-step chromatographic procedure, determination of the N-terminal amino acid sequence of purified bacteriocins by Edman degradation and a MALDI TOF/TOF tandem MS procedure permitted characterization of bacteriocins present in supernatants of producer cells.

**Conclusions:**

Enterococci was the largest LAB group with bacteriocinogenic activity isolated from griffon vulture feces. Among the isolates, *E. faecium* M3K31 has been identified as producer of enterocin HF (EntHF), a bacteriocin with remarkable antimicrobial activity against most evaluated *Listeria* spp. and of elevated interest as a natural food preservative. *E. faecium* M3K31 would be also considered a safe probiotic strain for use in animal nutrition.

**Electronic supplementary material:**

The online version of this article (doi:10.1186/s12866-016-0840-2) contains supplementary material, which is available to authorized users.

## Background

Lactic acid bacteria (LAB) are part of the gut microbiota of mammals and birds with an important role in environmental, food and clinical microbiology. Many LAB produce ribosomally synthesized antimicrobial peptides or bacteriocins which attract considerable interest as natural and nontoxic food preservatives [1], and as therapeutic agents for human and veterinary applications and in the animal production field [2, 3]. Bacteriocin-producing LAB are also attractive vectors for delivery of therapeutic peptides and proteins and as probiotics [4–6].

Most bacteriocins produced by LAB are synthesized as biologically inactive precursors or prepeptides containing an N-terminal extension that is cleaved off during export to generate their biologically active or mature form. They are generally divided into two main classes. Class I consists of the lanthionine-containing post-translationally modified bacteriocins or lantibiotics, while class II consists of the bacteriocins containing non-modified amino acids. The class II bacteriocins may be further subdivided into the pediocin-like (class IIa) bacteriocins, the two-peptide (class IIb) bacteriocins, the cyclic (class IIc) bacteriocins, and the non-pediocin-like one peptide linear (class IId) bacteriocins [7, 8]. However, additional subgroups have been suggested for leaderless peptides, circular bacteriocins, linear peptides derived from large proteins, and the glycosylated bacteriocins [9].

Environmental sources as well as wild and game animals are a powerful source of bacteriocin-producing LAB [10–12]. Griffon vultures (*Gyps fulvus* subspecies *fulvus*) belong to the Old World vultures group, a diverse mix of colonial cliff-nesting scavenger birds that play an essential ecological role as garbage collectors and recyclers. Their feeding habits are based almost exclusively on carrion, preferentially of mammals. Consequently, their gut microbiota may derive from ecological and evolutionary strategies for carrion exploitation [13]. Symbiotic relationships among animal hosts and bacteria that confer protection against pathogens are widespread in nature and are considered a driving force in evolution [14]. Therefore, griffon vultures might be considered as potential reservoirs of bacteriocin-producing LAB, with potential biotechnological applications. This work constitutes a first approach on the evaluation of the antimicrobial activity and safety aspects of bacteriocinogenic LAB isolated from griffon vulture feces.

## Methods

### Microbiological analysis, indicator strains and bacteriocinogenic assays

Fresh fecal samples from griffon vultures (*Gyps fulvus* subspecies *fulvus*) were collected from the Parque Natural del Alto Tajo (Molina de Aragón, Guadalajara, Spain), and placed into sterile disposable plastic tubes at 4 °C. Samples were 10-fold diluted in sterile peptone water (Oxoid Ltd., Basingstoke, UK) and homogenized in a stomacher. Aliquots from decimal dilutions of the homogenates were spread on duplicate plates of (i) Triptycase Soya Agar (TSA; Oxoid) at 37 °C, 48 h for aerobic mesophilic counts, (ii) on de Man, Rogosa, and Sharpe (MRS) plates (Oxoid) at 37 °C, 48 h for growth of LAB, and (iii) on Slanetz and Bartley (SB) plates (Oxoid) and Kanamycin Aesculin Azide (KAA) plates with the kanamycin selective supplement (Oxoid) at 37 °C, 48 h for growth of the enterococci. The direct antimicrobial activity of randomly selected isolates was screened by the stab-on-agar test (SOAT) [15] against three Gram-positive and four Gram-negative indicator bacteria. Next, cell-free supernatants of isolates producing halos of inhibtion larger than 7 mm or antimicrobial activity against, at least, three Gram positive indicator strains were evaluated for their antimicrobial activity by an agar diffusion test (ADT), against a larger number of Gram-positive indicators. Finally, the most active isolates were also evaluated for the antimicrobial activity of their supernatants by a microtiter plate assay (MPA) [15] against *Listeria* spp. strains. In the MPA test, one bacteriocin unit (BU) is defined as the reciprocal of the highest dilution of the bacteriocin causing 50 % growth inhibition (50 % of the turbidity of the control culture without bacteriocin). Supernatants were subjected to proteolytic treatment with proteinase K (Sigma-Aldrich GmbH, Madrid, Spain), at 10 mg/ml for 37 °C during 2 h, to ascertain the protein nature of their antagonistic activity. After proteinase inactivation by heat treatment (100 °C, 10 min), samples were assayed for residual antimicrobial activity by ADT, as described above, using *Pediococcus damnosus* CECT4797 as the indicator microorganism. Strains with antimicrobial activity in their supernatants and susceptible to proteinase treatment were considered Bac^+^ and selected for further characterization. Indicator strains and specific bacterial growth conditions used in this study are shown in Table 1.

**Table 1:** Indicator species and specific bacterial growth conditions used in this study

Organism	Origin/Reference^a^	Growth conditions	
		Medium	T (°C)
Gram-positive			
*Enterococcus faecalis*			
BFE 1071	[54]	MRS	37
DAC9	DNBTA	MRS	37
DBH9	DNBTA	MRS	37
DBH18	DNBTA	MRS	37
INIA 4	INIA	MRS	37
JH2-2	HRC	MRS	37
P4	IFR	MRS	37
V583	LMG	MRS	37
*Enterococcus faecium*			
L50	DNBTA	MRS	37
M3K31	This work	MRS	37
P13	DNBTA	MRS	37
T136	DNBTA	MRS	37
*Enterococcus hirae* DCH5	DNBTA	MRS	37
*Lactobacillus sakei* 2714	NCDO	MRS	37
*Lactococcus lactis* BB24	DNBTA	MRS	37
*Listeria grayii 931*	CECT	BHI	37
*Listeria innocua* 910	CECT	BHI	37
*Listeria ivanovii* 913	CECT	BHI	37
*Listeria seeligeri* 917	CECT	BHI	37
*Listeria welshimeri* 919	CECT	BHI	37
*Listeria monocytogenes* 911	CECT	BHI	37
*Listeria monocytogenes* 935	CECT	BHI	37
*Listeria monocytogenes* 936	CECT	BHI	37
*Listeria monocytogenes* 939	CECT	BHI	37
*Listeria monocytogenes* 4031	CECT	BHI	37
*Listeria monocytogenes* 4032	CECT	BHI	37
*Pediococcus damnosus* 4797	CECT	MRS	32
*Pediococcus pentosaceus* FBB61	ATCC	MRS	32
Gram-negative			
*Aeromonas salmonicida* 3776	LMG	TSB	28
*Campylobacter jejuni* 33560	ATCC	BHI + 1 % horse serum	37
*Campylobacter jejuni* 11168	NCTC	BHI + 1 % horse serum	37
*Yersinia ruckeri* 3279	LMG	TSB	28

^a^Abbreviations as: *ATCC* American Type Culture Collection, VA, USA, *CECT* Colección Española de Cultivos Tipo, Valencia, Spain, *DNBTA* Departamento de Nutrición, Bromatología y Tecnología de los Alimentos, Facultad de Veterinaria, Universidad Complutense de Madrid, Madrid, Spain, *HRC* Servicio de Microbiología, Hospital Universitario Ramón y Cajal, Instituto Ramón y Cajal de Investigaciones Sanitarias (IRYCIS), Madrid, Spain, *IFR* Institute of Food Research, Norwich, UK, *INIA* Instituto Nacional de Investigación y Tecnologıía Agraria y Alimentaria, Madrid, Spain, *LMG* Laboratorium voor Microbiologie, University of Ghent, Ghent, Belgium, *NCDO*, National Collection of Dairy Organisms, Aberdeen, Scotland, UK; *NCTC* National Collection of Type Type Cultures, Salisbury, UK

### PCR analysis, DNA sequencing and other DNA manipulations

PCR amplifications were performed from total bacterial DNA obtained using the InstaGene matrix (Bio-Rad laboratories Inc., Hercules, CA, USA) in 25 or 50 μl reaction mixtures containing MyTaq mix buffer (Bioline Reagents Ltd., London, UK), 0.7 μM of each primer and 1 μl of purified DNA. Oligonucleotide primers were obtained from Sigma Genosys Ltd. (Cambridge, UK). Samples were subjected to PCR amplification in an Eppendorf Mastercycler thermal cycler (Eppendorf, Hamburg, Germany). When needed, the resulting PCR fragments were purified using the NucleoSpin Extract II kit (Macherey-Nagel, Düren, Germany) and sequenced at the Unidad de Genómica (Parque Científico de Madrid, Facultad de Ciencias Biológicas, Universidad Complutense de Madrid, Spain).

### Genus and species identification, and detection of bacteriocin structural genes and potential virulence factors

From the 332 LAB isolates showing direct antimicrobial activity, 95 of them were taxonomically identified by PCR amplification and sequencing of genes encoding 16S rRNA (16S *rDNA*) with primers plb16/mlb16 [16], and the gene encoding superoxide dismutase (*sodA*) with primers d1/d2 [17]. Genus and species identification was performed by nucleotide BLAST analysis using the NCBI platform.

The presence of bacteriocin structural genes of previously described bacteriocins, was evaluated in 28 LAB isolates with the highest antimicrobial activity in their supernatants. A total of 21 bacteriocin structural genes were analysed including (i) the pediocin-like class IIa bacteriocins avicin A (AviA), bacteriocin 31 (Bac31), bacteriocin MC4-1 (BacMC4-1), enterocin A (EntA), enterocin SE-K4 (EntSE-K4), enterocin P (EntP), hiracin JM79 (HirJM79), mundticin L (MunL) and pediocin PA-1 (PedA-1); (ii) the (ii) two-peptide class IIb bacteriocins enterocin 1071A-1071B (Ent1071A-Ent1071B) and enterocin XA-XB (EntXA-EntXB); (iii) the circular class IIc bacteriocin enterocin AS-48 (EntAS-48); (iv) the class IId non-pediocin-like one peptide linear bacteriocins including the leaderless bacteriocins enterocin L50 (EntL50A-EntL50B), enterocin JS (EntJSA-EntJSB) and enterocin Q (EntQ), and other small heat-stable linear bacteriocins such as enterocin B (EntB), enterocin 96 (Ent96), enterocin IT (EntIT), enterocin V583 (EntV583) and brevicin 925A (BreB); as well as the large bacteriolysin enterolysin A (EnlA). The specific oligonucleotide primers, PCR conditions, positive control strains and references concerning each of the bacteriocin structural genes, are shown in Additional file 1: Table S1.

The presence of genes coding potential virulence factors was evaluated in the nine most active bacteriocinogenic *E. faecalis* isolates, by using primer pairs and PCR conditions designed for detection of genes *cylL*_L_–*cylL*_S_ (cytolysin precursor), *cylM* (postranslational modification of cytolysin), *cylB* (transport of cytolysin), *cylA* (activation of cytolysin), *ace* (adhesin to collagen), *agg* (aggregation substance), *esp* (enterococcal surface protein), *efaAfm* and *efaAfs* (cell wall adhesins of *E. faecium* and *E. faecalis*, respectively) and *gelE-sprE* and *sprE* (gelatinase and serine protease E), as previously described [10, 18].

### Safety assessment of *E. faecium* M3K31

The safety assessment of the *E. faecium* M3K31 isolate was determined according to guidelines established by the European Food Safety Authority (EFSA) [19], including the evaluation of (i) ampicillin resistance, (ii) determination of *esp*, a putative glycosyl hydrolase (*hly*_*Efm*_) and identification of the insertion sequence *IS16* [20].

### Production of gelatinase, caseinolytic and hemolytic activity, and antibiotic susceptibility testing

For production of gelatinase, single colonies of the most active nine bacteriocinogenic *E. faecalis* isolates, previously grown on MRS agar (Oxoid), were streaked onto Todd-Hewitt agar (Oxoid) containing 30 g of gelatin (Oxoid) per liter, grown overnight at 37 °C, and placed at 4 °C for 5 h before examination of zones of turbidity around the colonies. The caseinolytic activity of the isolates was evaluated by streaking the colonies onto TSA agar (Oxoid) containing 1.5 % bovine skim milk powder (Oxoid) and overnight growth at 37 °C. A clear zone of hydrolysis within 24 h of growth was considered positive. For investigation of their haemolytic activity, the strains streaked on Columbia agar supplemented with 5 % (v/v) horse blood (COH, BioMérieux, Madrid, Spain) were grown at 37 °C for 1 to 2 days. Haemolysis was evidenced by the formation of clear zones surrounding the colonies on blood agar plates. The antibiotic susceptibility of the 27 selected enterococci with the highest antimicrobial activity in their supernatants was determined by overlaying antibiotic-containing disks (Oxoid) on the Diagnostic Sensitivity Test Agar (Oxoid), following the Clinical and Laboratory Standards Institute (CLSI) guidelines [21]. The antibiotics tested were ampicillin (10 μg), chloramphenicol (30 μg), ciprofloxacin (5 μg), erythromycin (15 μg), gentamicin (120 μg), nitrofurantoin (300 μg), norfloxacin (10 μg), penicillin G (10 IU), rifampicin (5 μg), teicoplanin (30 μg), tetracycline (30 μg), and vancomycin (30 μg). Inhibition zone diameters were measured after overnight incubation of the plates at 37 °C. Resistance phenotypes were recorded as recommended by the CLSI [21]. *E. faecalis* ATCC29212 and *Staphylococcus aureus* ATCC25923, were used as control strains.

### Enterobacterial repetitive intergenic consensus sequences (ERIC-PCR) and multilocus sequence typing (MLST) analysis

The clonal relationship among the 9 *E. faecalis* and 14 *E. faecium* isolates from griffon vultures feces with the highest antimicrobial activity in their supernatants and from other enterococci from food, environmental and clinical origin was evaluated by ERIC-PCR, as previously described [20]. The resulting patterns were interpreted after constructing dendrograms using the unweighted-pair group method with arithmetic mean (UPGM) and the similarity on the Dice’s coefficient, analyzed with the Phoretix v5.0 software (Nonlinear Dynamics Ltd., UK). For MLST analysis, purified genomic DNA from selected enterococcal isolates was used for PCR-amplification of internal fragments of seven housekeeping genes, as previously described [22]. The resulting PCR products were purified with an ExoSAP-IT PCR clean up reagent (USB Europe GmbH, Staufen, Germany) and sequenced in a ABI Prism 377 automated sequencer (Applied Biosystems, Foster City, CA, USA) at the Servicio de Microbiología, Hospital Universitario Ramón y Cajal, and Instituto Ramón y Cajal de Investigaciones Sanitarias (IRYCIS), Madrid (Spain). Clusters of related sequence types (STs) were grouped into clonal complexes (CCs) by using eBURST (http://www.mlst.net).

### Purification of bacteriocins

The peptides responsible of the antimicrobial activity of *E. faecium* M1M10, *E. faecium* M3K31 and *E. faecium* T136 (producer of EntA) were purified to homogeneity by using a multichromatographic procedure, as previously described [23, 24]. Briefly, 1-L cultures of supernatants from the early stationary phase were subjected to precipitation with ammonium sulphate (50 %, w/v), desalted by gel filtration (PD columns) and further subjected to cation-exchange (SP Sepharose Fast Flow) and hydrophobic-interaction (Octyl-Sepharose CL-4B) chromatographies, followed by reverse phase chromatography (PepRPC HR 5/5) in a fast-protein liquid chromatography system (ÄKTA, RP-FPLC). The most active fractions from the last chromatographic step were combined and rechromatographied on the reverse-phase column to obtain the purified bacteriocin. All the chromatographic columns and equipment were from GE Healthcare (Madrid, Spain).

### Mass spectrometry analysis and amino acid sequencing

Purified peptide fractions from the last RP-FPLC step were subjected to matrix-assisted laser desorption–ionization time-of-flight (MALDI-TOF) mass spectrometry (MS) at the Unidad de Proteómica, Universidad Complutense de Madrid (Madrid, Spain). MALDI-TOF MS analyses were performed in a 4800 Proteomics Analyzer MALDI-TOF/TOF mass spectrometer (Applied Biosystems, Framingham, MA, USA), operated in 1 KV reflector mode. All mass spectra were calibrated externally using a standard peptide mixture (AB Sciex, Foster City, CA, USA).

MALDI TOF/TOF tandem mass spectrometry (MS) was used to determine the partial amino acid sequence of the purified peptide produced by *E. faecium* M3K31. Acquisition of the MS data was performed on an Ultraflex MALDI-TOF/TOF (Bruker Daltonics Inc. Billerica, MA, USA) instrument operated in reflection mode with delayed extraction, at the Proteomics Core Facility of the Norwegian University of Life Sciences, Ås (Norway). MS/MS spectra of selected peptides were recorded using the LIFT ion optics of the mass spectrometer. Recorded spectra were processed in flexAnalysis software (v3.3, Bruker Daltonics) and mass lists submitted to database searches (via BioTools software, v 3.2, Bruker Daltonics) were performed using an in-house Mascot server (v.2.1). Manual annotation of MS/MS spectra (*de novo* sequencing) was performed in flexAnalysis. The sequences generated were searched against NCBI/taxonomy *Firmicutes* using protein BLAST.

For N-terminal amino acid sequencing, the purified peptide from *E. faecium* M1M10 was subjected to automatic Edman degradation and sequence on polyvinylidene difluoride membranes (PVDF) in a Procise 494 HT Sequencing System (Applied Biosystems Inc., Foster City, CA, USA) at the Centro de Investigaciones Biológicas (CIB, Madrid, Spain).

## Results

### Identification of isolates with antimicrobial activity

In this study, 406 randomly selected isolates from griffon vultures feces grown on MRS plates, 418 isolates grown in SB plates and 100 isolates grown on KAA plates, were evaluated for their direct antimicrobial activity (SOAT) against 3 Gram-positive indicators (*Pediococcus damnosus* CECT4797*, Lactococcus lactis* BB24 and *Listeria innocua* CECT910) and 4 Gram-negative bacteria (*Yersinia ruckeri* LMG3279*, Aeromonas salmonicida* LMG3776, *Campylobacter jejuni* ATCC33560 and *Campylobacter jejuni* NCTC11168). From the 924 LAB evaluated, 332 isolates (36 %) showed direct antimicrobial activity against, at least, one of the Gram-positive bacterial indicators tested. However, no evidence for direct antimicrobial activity was shown against any of the four Gram-negative bacteria tested, including the two *C. jejuni* strains. From this initial screening, 95 LAB isolates with the largest halos of inhibition were identified by PCR amplification and sequencing of genes encoding 16S *rDNA* and *sodA*. From these results 38 *E. faecium* (40 %), 30 *E. faecalis* (31 %) 1 *E. hirae* (1 %), 1 *E. mundtii* (1 %), 5 *Lactobacillus brevis* (5 %) and 1 *Lactobacillus plantarum* (1 %) strains were identified. The remaining 19 isolates were identified as *Enterococcus* spp. (20 %).

From the above cited 95 LAB isolates, a set of 28 isolates comprising 9 *E. faecalis*, 16 *E. faecium*, 1 *E. hirae*, 1 *E. mundtii* and 1 *Lb. brevis* were tested for antimicrobial activity in their supernatants by an agar diffusion test (ADT). Among the *E. faecalis* isolates, *E. faecalis* M2M6 and *E. faecalis* M3M42 were active against a number of Gram-positive bacteria, but none of the isolates were active against any of the Gram-negative indicators tested (Table 2). When supernatants from *E. faecium*, *E. hirae*, *E. mundtii* and *Lb. brevis* were tested for their antimicrobial activity most *E. faecium* and the *Lb. brevis* isolate were active against most Gram-positive indicators, with no antagonistic activity observed for *E. hirae* M4S3. It is noteworthy to observe the high antimicrobial activity of *E. faecium* M3K31 against most of the bacterial Gram-positive indicators tested. However, none of the enterococcal and the *Lb. brevis* supernatants evaluated were active against *C. jejuni* ATCC33560 (Table 3). Supernatants of the most antagonistic LAB isolates (3 *E. faecalis*, 5 *E. faecium*, 1 *E. mundtii*, and 1 *Lb. brevis*) were also evaluated against 11 *Listeria* spp. by a microtiter plate assay (MPA). The supernatant of *E. faecium* M3K31 was remarkably active against most of the *Listeria* spp. evaluated (Table 4).

**Table 2:** Antimicrobial activity^a^ of supernatants from selected *E. faecalis* isolates

Isolate	Indicator microorganisms^b^														
	*E. faecalis*								*E. faecium*		*P. damnosus* 4797	*L. lactis* BB24	*Y. ruckeri* 3276	*A. salmonicida* 3276	*C. jejuni* 33560
	BFE 1071	DAC9	DBH9	DBH18	INIA4	JH2-2	P4	V583	T136	L50					
*E. faecalis*															
AS10	-	-	-	-	-	-	-	9.7	-	-	7.2	-	-	-	-
M1M32	-	-	-	-	-	-	-	7.4	-	-	8.9	-	-	-	-
M1S19	-	-	-	-	-	-	-	-	-	-	7.2	-	-	-	-
M1S20	-	-	-	-	-	-	-	-	-	-	6.9	-	-	-	-
M2M6	9.2	14.1	11.5	-	-	-	9.6	11.1	12.0	-	16.8	-	-	-	-
M2M39	-	-	-	-	-	-	-	-	-	-	8.2	-	-	-	-
M3M42	9.4	13.6	10.0	-	-	-	8.6	8.3	12.6	-	10.3	-	-	-	-
M3S1	-	-	-	-	-	-	-	-	-	-	6.7	-	-	-	-
PM2-13	-	-	-	-	-	-	-	8.5	-	-	12.2	-	-	-	-

^a^Antimicrobial activity determined by ADT. Results as the diameter of the inhibition halos in millimeters (mm). (−) Antimicrobial activity not detected. Most of the data are means from two independent determinations in triplicate

^b^Source of indicator microorganisms indicated in Table 1

**Table 3:** Antimicrobial activity^a^ of supernatants from isolated *E. faecium* and other lactic acid bacteria (LAB)

Isolate	Indicator microorganisms^b^															
	*E. faecalis*				*E. faecium*				*E. hirae* DCH5	*L. lactis* BB24	*L. sakei* 2714	*L. innocua* 910	*L. monocytogenes* 4032	*P. damnosus* 4797	*P. pentosaceus* FBB61	*C. jejuni* 33560
	DBH18	INIA4	JH2-2	V583	L50	M3K31	P13	T136								
*E. faecium*																
AS 41	11.5	7.6	11.7	12.8	15.2	12.7	13.4	-	13.6	-	17.9	12.1	13.4	20.4	-	-
BS15	12.2	7.3	11.3	14.6	14.2	14.4	14.0	-	14.0	-	18.9	12.7	13.5	21.3	9.3	-
CS14	11.5	7.1	12.4	12.6	16.9	14.6	15.0	-	14.2	-	18.8	12.1	12.4	22.9	8.6	-
CS46	11.7	8.6	13.5	13.6	16.8	16.9	14.4	-	14.5	-	19.2	11.6	12.5	23.0	8.7	-
M1M10	10.0	-	11.3	13.0	15.1	13.0	14.8	-	14.3	-	15.2	12.4	12.3	21.0	-	-
M1M26	9.4	-	11.8	13.3	15.5	12.6	14.1	-	14.6	-	15.2	11.8	11.6	20.9	-	-
M2M33	-	-	-	-	-	-	-	-	-	-	8.2	-	-	12.2	-	-
M2S31	9.4	-	11.1	11.5	14.2	13.8	14.4	-	-	-	14.5	11.3	12.4	16.8	-	-
M3M31	11.0	7.0	11.8	13.9	16.4	14.6	14.9	-	14.1	-	15.8	12.3	13.2	20.9	9.7	-
M3M32	11.6	8.8	12.8	14.9	16.1	17.9	16.0	-	15.1	-	8.4	11.9	13.0	22.9	10.3	-
M3K31	16.3	15.5	14.3	16.4	16.9	-	18.7	17.8	16.8	18.2	18.0	15.3	17.0	26.4	19.2	-
M4M2	-	-	-	-	-	-	-	-	-	-	-	-	-	10.1	-	-
PM1-27	11.2	-	10.7	14.0	13.9	12.7	15.8	-	-	-	19.0	11.1	13.6	17.2	-	-
PM1-32	13.0	7.5	11.7	15.5	15.0	12.9	15.5	-	-	-	18.3	11.7	13.9	17.3	10.3	-
PM1-36	12.2	-	11.8	14.5	13.9	12.1	14.5	-	-	-	18.9	12.5	13.8	17.6	9.1	-
PM1-47	13.1	7.8	12.4	14.0	15.6	13.5	15.2	-	-	-	18.5	11.6	12.9	19.5	9.6	-
*E. hirae*																
M4S3	-	-	-	-	-	-	-	-	-	-	-	-	-	-	-	-
*E. mundtii*																
M2M33	-	-	-	-	-	-	-	-	-	-	8.2	-	-	12.2	-	-
*L. brevis*																
PM1-26	11.1	-	10.0	14.6	15.1	12.5	14.0	-	-	-	17.0	11.1	12.6	16.0	-	-

^a^Antimicrobial activity determined by ADT. Results as the diameter of the inhibition halos in millimeters (mm). (−) Antimicrobial activity not detected. Most of the data are means from two independent determinations in triplicate

^b^Source of indicator microorganisms indicated in Table 1

**Table 4:** Antimicrobial activity^a^ of supernatants from selected LAB against *Listeria* spp.^b^

Strain	*L. grayii* 931	*L. innocua* 910	*L. ivanovii* 913	*L. seeligeri* 917	*L. welshimeri* 919	*L. monocytogenes*					
						911	935	936	939	4031	4032
*E. faecalis*											
M2M6	0.50	0.06	0.05	0.12	0.06	0.06	0.05	0.06	0.06	0.06	0.06
M3M42	0.49	0.06	0.05	0.12	0.06	0.06	0.06	0.06	0.11	0.57	0.06
PM2-13	0.10	0.13	1.05	0.29	0.27	0.22	0.15	0.29	0.15	0.15	0.27
*E. faecium*											
M1M10	9.1	2.6	16.3	2.2	4.3	34.5	8.5	18.6	3.9	3.5	16.3
M3M31	3.6	3.8	97.9	8.3	19.8	4.1	7.9	18.3	8.2	5.1	12.5
M3M32	3.8	1.9	288.4	35.0	20.1	7.7	7.8	34.9	14.6	4.2	34.7
M3K31	27.6	30.7	1.1 × 10^6^	3.0 × 10^5^	1.7 × 10^3^	12.2	1.1 × 10^3^	6.0 × 10^3^	3.9 × 10^4^	1.7x10^6^	8.6 × 10^6^
PM1-27	1.9	2.3	9.3	7.5	3.7	4.8	3.8	8.7	4.4	1.9	8.4
*E. mundtii*											
M2M33	NA	0.24	0.23	0.17	0.19	0.17	8.74	0.07	0.08	0.08	0.16
*L. brevis*											
PM1-26	3.8	1.9	29.1	4.4	4.7	5.0	3.8	9.4	4.4	1.8	8.6

^a^Antimicrobial activity measured by MPA and expressed as 10^3^ × BU/mL. NA, No antimicrobial activity detected. Most of the data are means from two independent determinations in triplicate

^b^Source of indicator microorganisms indicated in Table 1

### Evaluation of bacteriocin structural genes

Purified genomic DNA of the 28 selected LAB isolates was subjected to PCR amplification to determine the presence of structural genes coding for 21 previously described bacteriocins. All isolates encoded, at least, one described bacteriocin gene except *E. hirae* M4S3. From the *E. faecalis* isolates evaluated all of them encoded *entV583*, 6 isolates *enlA*, 5 isolates *ent1071A-ent1071B*, 2 isolates *ent96* and 2 more isolates encoded *entJSA-entJSB*. Five bacteriocin-producing genes (for *ent*96, *ent*1071A-*ent*1071B, *ent*JSA-*ent*JSB, *ent*V583, *enl*A) were detected in *E. faecalis* M2M6 and three (for *ent*1071A-*ent*1071B, *ent*V583, *enl*A) in *E. faecalis* M1S20. Among the *E. faecium* isolates, the *entA* gene was detected in 13 (86.6 %) out of the 15 evaluated isolates and, associated with the *ent*B gene, in 7 (53.8 %) of the isolates (Table 5). The *ent96, entK4, entP and entXA-entXB* and *hirJM79* bacteriocin-producing genes were shown to have a lower incidence (3.5 to 17.8 %) in the evaluated isolates while the *avi*A, *bac*31, *bac*MC4, *ent*AS48, *ent*IT, *ent*L50A-*ent*L50B, *ent*Q and *mun*L structural genes, could not be detected in any of the evaluated isolates. *E. faecium* M3K31 was shown to encode only the *entP* gene (Table 5).

**Table 5:** PCR amplification of bacteriocin structural genes from selected bacteriocinogenic lactic acid bacteria (LAB) isolates

Isolate	*aviA*	*bac31*	*bacMC4*	*breB*	*ent96*	*ent1071A-ent1071B*	*entA*	*entAS-48*	*entB*	*entIT*	*entJSA-entJSB*	*entK4*	*entL50A-entL50B*	*entP*	*entQ*	*entV583*	*entXA-entXB*	*enlA*	*hirJM79*	*munL*	*pedA-1*
*E. faecalis*																					
AS10	-	-	-	-	-	-	-	-	-	-	-	-	-	-	-	+	-	-	-	-	-
M1M32	-	-	-	-	-	+	-	-	-	-	-	-	-	-	-	+	-	+	-	-	-
M1S19	-	-	-	-	-	+	-	-	-	-	-	-	-	-	-	+	-	+	-	-	-
M1S20	-	-	-	-	-	+	-	-	-	-	-	-	-	-	-	+	-	+	-	-	-
M2M6	-	-	-	-	+	+	-	-	-	-	+	-	-	-	-	+	-	+	-	-	-
M2M39	-	-	-	-	+	+	-	-	-	-	-	-	-	-	-	+	-	+	-	-	-
M3M42	-	-	-	-	-	-	-	-	-	-	+	-	-	-	-	+	-	-	-	-	-
M3S1	-	-	-	-	-	-	-	-	-	-	-	-	-	-	-	+	-	+	-	-	-
PM1-27	-	-	-	-	-	-	-	-	-	-	-	-	-	-	-	+	-	-	-	-	-
PM2-13	-	-	-	-	-	-	-	-	-	-	-	-	-	-	-	+	-	-	-	-	-
*E. faecium*																					
AS41	-	-	-	-	-	-	+	-	+	-	-	-	-	-	-	-	+	-	-	-	-
BS15	-	-	-	-	-	-	+	-	+	-	-	-	-	-	-	-	-	-	-	-	-
CS14	-	-	-	-	-	-	+	-	+	-	-	-	-	-	-	-	-	-	-	-	-
CS46	-	-	-	-	-	-	+	-	+	-	-	-	-	-	-	-	+	-	-	-	-
M1M10	-	-	-	-	+	-	+	-	+	-	-	+	-	-	-	-	+	-	-	-	-
M1M26	-	-	-	-	-	-	+	-	-	-	-	-	-	-	-	-	-	-	+	-	-
M2S31	-	-	-	-	-	-	+	-	+	-	-	-	-	-	-	-	-	-	-	-	-
M2M31	-	-	-	-	-	-	+	-	+	-	-	-	-	-	-	-	+	-	-	-	-
M3M32	-	-	-	-	-	-	+	-	-	-	-	-	-	-	-	-	+	-	-	-	-
M3K31	-	-	-	-	-	-	-	-	-	-	-	-	-	+	-	-	-	-	-	-	-
M4M2	-	-	-	-	-	-	-	-	-	-	-	-	-	-	-	-	-	-	-	-	-
PM1-27	-	-	-	-	-	-	+	-	-	-	-	-	-	-	-	-	-	-	-	-	-
PM1-36	-	-	-	-	-	-	+	-	-	-	-	-	-	-	-	-	-	-	+	-	-
PM1-37	-	-	-	-	-	-	+	-	-	-	-	-	-	-	-	-	-	-	-	-	-
PM1-47	-	-	-	-	-	-	+	-	-	-	-	-	-	-	-	-	-	-	-	-	-
*E. hirae*																					
M4S3	-	-	-	-	-	-	-	-	-	-	-	-	-	-	-	-	-	-	-	-	-
*E. mundtii*																					
M2M33	-	-	-	-	-	-	-	-	+	-	-	+	-	-	-	-	-	-	-	-	-
*L. brevis*																					
PM1-26	-	-	-	+	-	-	-	-	-	-	-	-	-	-	-	-	-	-	-	-	-

### Potential virulence factors, antibiotic susceptibility and hemolytic activity

When the previously selected 9 bacteriocinogenic *E. faecalis* isolates were evaluated for potential virulence factors the presence of the *cylL*_*L*_*-cylL*_*S*_ genes, encoding the two peptide cytolysin (hemolysin-bacteriocin) precursor, was detected in three isolates. However, only *E. faecalis* PM2-13 showed the presence of the *cylLMAB* genes for expression of cytolysin. Accordingly, only *E. faecalis* PM2-13 showed β-hemolytic activity when streaked on blood agar plates. Only two *E. faecalis* isolates encoded *agg* but none encoded the *esp* gene. Most *E. faecalis* isolates encoded *ace*, *gelE* and *sprE* but *E. faecalis* M1S9 and *E. faecalis* M3S1 did not encode any of the three cited genes (Table 6). All the evaluated *E. faecalis* strains hydrolysed gelatin and bovine casein, except those isolates not encoding *gelE-sprE*.

**Table 6:** PCR amplification of genes related to potential virulence in *E. faecalis*

Isolate	Virulence determinants										
	*ace*	*agg*	*cylA*	*cylB*	*cylL*_*L*_ *-cylL*_*S*_	*cylM*	*efaAfm*	*efaAfs*	*esp*	*gelE*	*sprE*
*E. faecalis*											
AS10	+	-	-	-	-	-	-	+	-	+	+
M1M32	+	+	-	-	-	+	-	+	-	+	+
M1S19	-	+	+	+	+	-	-	+	-	-	-
M1S20	+	-	-	-	-	-	-	+	-	+	+
M2M6	+	-	-	-	-	-	-	+	-	+	+
M2M39	+	-	-	-	-	-	-	+	-	+	+
M3M42	+	-	-	-	-	+	-	+	-	+	+
M3S1	-	-	+	+	+	-	-	+	-	-	-
PM2-13	+	-	+	+	+	+	-	+	-	+	+

Antibiotic susceptibility of the 27 selected bacteriocinogenic enterococci revealed that all of them (100 %) were resistant to at least one of the tested antibiotics. Furthermore, 85 % of the isolates were resistant to rifampicin, 77 % to tetracycline, 50 % to erythromycin, 44 % to cyprofloxacin, 29 % to chloramphenicol, 28 % to nitrofurantoin, 29 % to gentamycin and ampicillin, 25 % to penicillin, and 7 % to vancomycin. However, *E. faecalis* M3M42 and *E. faecium* M3K31 were only resistant to rifampicin while *E. faecalis* M2M6 was sensitive to all antibiotics tested. All isolates were sensitive to teicoplanin. The genotypic evaluation of the antibiotic resistance profile of the enterococci evaluated was not pursued in this study.

### Safety assessment of *E. faecium* M3K31

The sensitivity of *E. faecium* M3K31 to ampicillin resulted in a minimum inhibitory concentration (MIC) ≤ 2 mg/L and, thus, susceptible to ampicillin according to EFSA guidelines [19]. This isolate also showed the absence of the virulence markers *esp*, *hyl*_*Efm*_ and *IS16*.

### Molecular genotyping of selected enterococcal strains by ERIC-PCR and MLST analysis

**Fig. 1:**
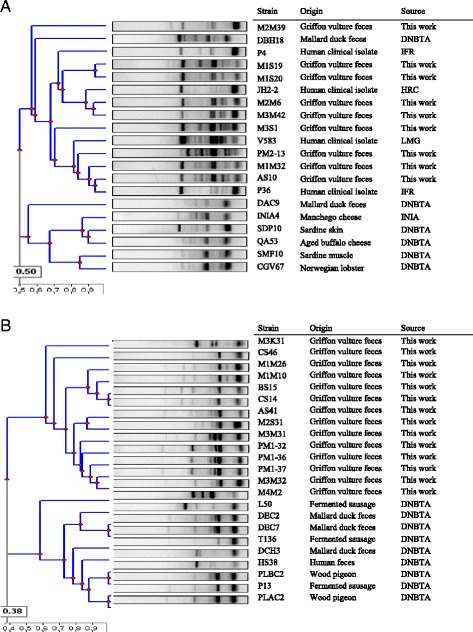
Dendrogram of ERIC-PCR showing the relatedness amongst the *E. faecalis* (**a**) and *E. faecium* (**b**) isolates from griffon vulture feces and enterococcal isolates from other food, environmental and clinical origin. Source abbreviations as indicated in Table 1

The ERIC-PCR fingerprints of the nine most antagonistic *E. faecalis* strains isolated from griffon vultures feces and those from other *E. faecalis* strains from different food, environmental and clinical origin, revealed 2 different clusters (50 % similarity). The first cluster included all the isolates from griffon vulture feces and other *E. faecalis* strains, mostly from human clinical origin. The second cluster included only *E. faecalis* strains from different origins (Fig. 1a). The ERIC-PCR dendrogram showing the genetic relatedness between *E. faecium* isolates from different origins could be also divided into two clusters. One cluster contained 13 out of the 14 most antagonistic *E. faecium* isolates from griffon vultures showing 60 % similarity. The second cluster grouped all the *E. faecium* isolates from different origins including *E. faecium* M4M2, isolated from griffon vultures. Among the griffon vulture isolates only *E. faecium* BS15 and *E. faecium* CS14 showed an almost identical genotype (Fig. 1b).

Comparison of the resulting allelic profiles of *E. faecalis* M3M42 and M1S19 in the *E. faecalis* MLST database permitted their annotation as sequence types ST167 and ST76, respectively. On the other hand, *E. faecium* M1M10, *E. faecium* M3K31 and *E. faecium* PM1-27 were annotated as sequence types ST22, ST76 and ST670, respectively, in the *E. faecium* MLST database. Comparative eBURST analysis of the resulting sequence types (STs) permitted their location into clusters of related STs (Additional file 1: Figure S1).

### Purification of bacteriocins, mass spectrometry analysis and amino acid sequencing

**Fig. 2:**
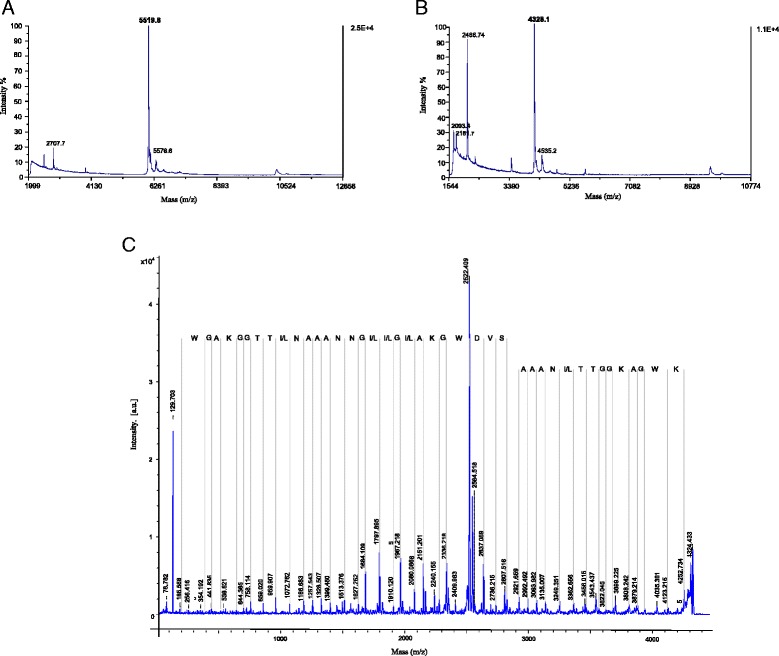
MALDI-TOF MS analysis of purified bacteriocins from *E. faecium* M1M10 (**a**) and *E. faecium* M3K31 (**b**). MALDI TOF/TOF tandem MS analysis of the purified bacteriocin from *E. faecium* M3K31 (**c**). The carboxy-terminal (y-ion series) shows the 27-residue peptide SVDWGKAIGIIGNNAAANLTTGGKAGW (top line) and the amino-terminal (b-series) shows the 14-residue peptide AAANLTTGGKAGWK (bottom line). Numbers indicate molecular mass in daltons

Two of the bacteriocinogenic enterococci isolated from griffon vulture feces, *E. faecium* M1M10 and *E. faecium* M3K31 with high antimicrobial activity in their supernatants and encoding several or a single bacteriocin structural gene, respectively, were selected for purification of the bacteriocins being produced. Purification of the antimicrobial activity of *E. faecium* M1M10, encoding five bacteriocin structural genes, permitted a 1397-fold increase of its specific activity and a 32 % recovery of the initial antimicrobial activity. MALDI-TOF MS of the purified peptide showed a major peak of 5519.8 Da, suggesting the peptide was purified to homogeneity (Fig. 2a). Furthermore, determination of the N-terminal amino acid sequence of the purified peptide by Edman degradation permitted identification of its 15 N-terminal amino acids as ENDHRMPNELNRPNN, which unambigously correspond to the N-terminal sequence of the enterocin B (EntB). However, EntB has a calculated theoretical molecular mass of 5465.2 Da. Purification of the antimicrobial activity of *E. faecium* M3K31, encoding the *entP* gene, permitted an 897-fold increase of its specific activity and a 11 % recovery of the initial antimicrobial activity. MALDI-TOF MS analysis of the purified peptide showed a major peak of 4328.1 Da (Fig. 2b). Furthermore, the *de novo* amino acid sequencing of the resulting peptide by MALDI TOF/TOF tandem MS permitted identification of the carboxy-terminal (y-ion series) 27-residue peptide SVDWGKAIGIIGNNAAANLTTGGKAGW and the amino-terminal (b-ion series) 14-residue peptide AAANLTTGGKAGWK (Fig. 2c), identical to the C-terminal amino acid sequence of enterocin HF (EntHF) (GenBank accession numbers P86183 and KJ442693), and with a theoretical molecular mass of 4330.9 Da. Primers devised from the nucleotide sequence of EntHF from GenBank accession number KJ442693 and PCR amplifications and sequencing of the resulting fragments, confirmed that *E. faecium* M3K31 encoded *entHF*.

### Antimicrobial activity of purified enterocin A (EntA) and enterocin HF (EntHF) against *Listeria* spp

The sensitivity of several *Listeria* spp. to purified EntA, produced by *E. faecium* T136 and a potent antilisterial class IIa bacteriocin and to EntHF, produced by *E. faecium* M3K31, is shown in Table 7. Purified EntHF showed a higher specific antimicrobial activity against *Listeria* spp. than purified EntA. Furthermore, *L. ivanovii* CECT913 was the most sensitive indicator strain for both bacteriocins.

**Table 7:** Antimicrobial activity^a^ of chromatographically purified enterocin HF and enterocin A produced by *E. faecium* M3K31 and *E. faecium* T136, respectively, against *Listeria* spp. and *P. damnosus* CECT4797^b^

Purified bacteriocins	*L. grayii* 913	*L. innocua* 910	*L. ivanovii* 913	*L. seeligeri* 917	*L. welshimeri* 919	*L. monocytogenes*						*P. damnosus* 4797
						911	935	936	939	4031	4032	
Enterocin HF	1,168	4	8.3 × 10^9^	582	464	9	364	1,118	155	55	2,455	73
Enterocin A	8	9	19,000	10	5	10	17	8	10	2	14	5

Most of the data are means from two independent determinations in triplicate

^a^Antimicrobial activity measured by MPA and expressed as BU/ng of purified bacteriocin

^b^Source of indicator microorganisms indicated in Table 1

## Discussion

Among other features, most bacteria produce compounds that inhibit competing and pathogenic microorganisms, improving host health [7, 25]. Since griffon vultures feed regularly on carcasses from dead birds and mammals, it may be hypothesized that vultures could benefit themselves hosting bacteriocin-producing LAB to combat bacterial pathogens. In this study, 36 % of the evaluated LAB isolates showed direct antimicrobial activity. The enterococci comprised the largest LAB group with antimicrobial activity (91 %) with *E. faecium* (40 %) as the most identified antagonistic species. Enterococci with antimicrobial activity have been previously identified from LAB isolated from mammals, birds and fish [10–12], but not with this elevated percentage of isolation. Enterococci are also the most common species in cloacal and pharyngeal samples of Euroasian griffon vultures [26] and in Turkey and Black vultures whereas enterococcal symbionts living in the hoopoe (*Upua epops*) uropygial gland produce bacteriocins active against Gram-positive pathogens [27]. Thus, LAB with antimicrobial activity isolated from griffon vulture feces mainly contains bacteriocinogenic enterococci.

In this work none of the LAB isolated from griffon vultures showed antagonistic activity against any of the four Gram-negative indicator bacteria evaluated, including two *C. jejuni* strains. Since griffon vultures are not a reservoir for *Campylobacter* spp. [26, 28], perhaps bacteria different than LAB may inhibit or control their presence in this reservoir. Several nonribosomal lipopeptides produced *by Bacillus* and *Paenibacillus* spp. have shown antagonistic activity against *C. jejuni* [29]. Thus, these bacteria would be further evaluated for antimicrobial activity against *C. jejuni* when identified from animal and food reservoirs [30]. When selected LAB were evaluated for antimicrobial activity in their supernatants, the *E. faecalis* isolates (Table 2) were less active than *E. faecium* and other LAB isolates (Table 3), which has been observed from enterococci from other sources [10–12, 31]. But of interest is the high antimicrobial activity of *E. faecium* M3K31 against most bacterial indicators, including *L. lactis* BB24 (Table 3) and most of the *Listeria* spp. evaluated (Table 4).

In this study, most selected bacteriocinogenic LAB isolates encoded a variable number of bacteriocin structural genes (Table 5). However, the presence of structural bacteriocin-producing genes, either alone or in combination with others, seems to be common in bacteriocinogenic enterococci from human, animal, clinical, food, agricultural and environmental sources [10–12]. Furthermore, is also difficult to find a correlation between the number of bacteriocin structural genes and the antagonistic activity and antimicrobial spectrum of the isolates. It may be hypothesized that some bacteriocin-producing genes may be silent, that modifications in the promoter region may affect transcription, and that immunity/regulation/transport of the synthesized bacteriocin may also be impaired. The production of bacteriocins is also regulated by environmental factors such as the temperature [32].

The enterococci are LAB with a beneficial role in the sensory characteristics of fermented foods and have been successfully used as starter and adjunct cultures, and permitted as probiotics [33]. However, the enterococci are also gastrointestinal (GI) tract colonizers responsible of nosocomial and, to a lesser extent, community acquired infections [34]. *E. faecalis* harbours significantly more virulence determinants than *E. faecium* [10, 18] and, therefore, we screened the bacteriocinogenic *E. faecalis* isolates for presence of virulence determinants (Table 6). In this study, only *E. faecalis* PM2-13 amplified all genes for expression of cytolysin. Insertion/deletion events leading to truncated or absent *cyl* genes and possible sequence divergences may explain the difficulties to tackle genes involved in the production of active cytolysins. Most *E. faecalis* isolates encoded *ace*, *gelE* and *sprE*. Protease expression seems to be strain specific and not representative of clinical isolates although regulatory genes must be also active, to permit protease expression [35]. None of the *E. faecalis* evaluated encoded *esp* and this is considered a positive attribute since Esp is supposed to promote primary attachment to surfaces and escape from the immune system.

Enterococci are commonly resistant to macrolides, cephalosporins and tetracycline and often exhibit high-level resistance to gentamicin [36]. Vultures rely greatly on food which usually derives from medicated livestock that could lead vultures to the acquisition of antimicrobial-resistant bacteria, modification of the normal microbiota and acquisition of pathogenic bacteria. But, in this study several *E. faecalis* and *E. faecium* isolates did not show a large antimicrobial resistance. Moreover, *E. faecium* M3K31 was susceptible to ampicillin and did not encode the virulence markers *esp*, *hyl*_*Efm*_ and *IS16* being considered, according to EFSA guidelines [19], a safe probiotic for use in animal nutrition.

The virulence of the enterococci also reflects a selection for specific variants or clones with enhanced pathogenic potential [37]. The epidemiological typing of *E. faecalis* and *E. faecium* has mainly been performed by ERIC-PCR and MLST analysis. As expected, *E. faecalis* strains from griffon vulture feces showed a distinct genetic relatedness among them and from other strains from food, clinical and environmental origin when evaluated by ERIC-PCR (Fig. 1a). However, the *E. faecium* strains formed a more conserved, but also distinct group as compared to enterococcal isolates from different origins (Fig. 1b). These results suggest the reliability of ERIC-PCR for genotyping enterococci and, very likely, for their specific monitoring.

Previous MLST studies have demonstrated the association between specific clonal complexes and human nosocomial infections for *E. faecalis* [38, 39] and *E. faecium* [34]. Indeed, the majority of hospital-derived isolates of *E. faecalis* cluster in two clonal complexes, CC2 and CC9. However, in *E. faecium* the sequence types ST17, ST18, ST78 and ST192 which were previously designated clonal complex CC17, constitutes a hospital-associated clade genetically distinct from most commensal isolates [34, 37]. In this study, *E. faecalis* M3M42 and *E. faecalis* M1S19 were identified with the sequence types ST167 and ST76, respectively, none of them included into the clonal complexes CC2 and CC9 represented by sequence types ST6 and ST9, respectively (Additional file 1: Figure S1). In this context, *E. faecalis* M3M42 sensitive to most antibiotics, free of most virulence determinants and encoding *gelE-sprE* could be considered a potential strain for production of bioactive peptides with antihypertensive and antioxidant activity, during its growth on bovine skim milk [40, 41]. The MLST analysis of the *E. faecium* isolates showed that while *E. faecium* M1M10 (ST22) and *E. faecium* PM1-27 (ST670) remain close to strains from a hospital-associated clade the strain of *E. faecium* M3K31 (ST176) remains distant from those of the clonal complex CC17 and, thus, is considered a safe isolate (Additional file 1: Figure S1).

For bacteriocinogenic isolates the possibility exists for their antimicrobial activity being mediated by still unknown or not yet described bacteriocins or by regulation of the production of multiple encoded bacteriocins. In this study, MALDI-TOF MS analysis and N-terminal amino acid sequencing of the purified supernatant of *E. faecium* M1M10, encoding several bacteriocin structural genes, permitted the identification of a major peptide fragment (Fig. 2a) unambigously recognized as the N-terminal sequence of EntB [42]. The difference between the obtained and the calculated molecular mass of te EntB may be adscribed to still unknown modifications. Furthermore, the absence of other major peptide fragments in the purified activity of *E. faecium* M1M10, may imply that: (i) other encoded bacteriocins are produced in much lower concentrations, (ii) their structural genes remain silent and/or (iii) their production is regulated by still unknown mechanisms.

On the other hand, MALDI-TOF MS analysis and the *de novo* amino acid sequencing by MALDI TOF/TOF tandem MS of the major peptide from the purified supernatant of *E. faecium* M3K31 (Fig. 2b), encoding *entP* and with antimicrobial activity against *L. lactis* BB24, a LAB species with low or non-inhibitory activity by class IIa bacteriocins [9], permitted the identification of a peptide identical to the C-terminal amino acid sequence of the bacteriocin EntHF. The difference between the measured and the calculated molecular mass of EntHF, suggests the existence of a disulfide bond linking the two known cysteine residues in the molecule. In this context, mature EntHF is 91 % identical to mundticin KS/enterocin CRL35 [43, 44] and 90 % identical to mundticin L [45] and avicin A [46]. These bacteriocins display a high antilisterial activity, and enterocin CRL35 has even been postulated as a promising alternative agent for the in vivo prevention of *Listeria* spp. infections [47]. The absence of the bacteriocin EntP in the purified peptide fraction from *E. faecium* M3K31, imply that further studies on the regulation of the expression of bacteriocin structural genes in bacteriocinogenic enterococci would be pursued.

The specific antimicrobial activity of purified EntA and EntHF against *Listeria* spp. was higher for EntHF as compared to EntA (Table 7). Thus, although EntA is one of the most potent class IIa bacteriocins [48], EntHF is even more potent than EntA against *Listeria* spp. The determination of the three-dimensional (3D) structure of EntHF, produced by *E. faecium* M3K31, suggest that apparently the β-sheet-like N-terminal domain mediates initial binding to the target cell whereas the helix-containing C-terminal half penetrates into the hydrophobic core of target-cell membranes, docks to domains of specific protein receptors and causes dead of sensitive cells [49]. Modifications in the amino acid composition of the C-terminal end of class IIa bacteriocins may alter the manner and/or extent to which bacteriocins interact with putative or cognate receptors and even with cognate immunity proteins in the membrane of target cells [50, 51]. This would explain why the antimicrobial spectra of similar class IIa bacteriocins and the susceptibility of sensitive cells to a given bacteriocin, varies much more than would be expected from their known amino acid sequences. Accordingly, EntHF may be considered a bacteriocin of elevated potential biotechnological interest as a natural food preservative and a therapeutic antimicrobial agent for human and veterinary applications. Furthermore, because EntHF is a primary metabolite of a linear peptidic nature, it may be considered suitable for the development of novel bacteriocin quimeras with increased target specifity and antimicrobial activity by peptide bioengineering.

Within LAB, enterococci are increasingly used as probiotics. Most probiotics enhance intestinal barrier function, display immunomodulatory activity and exert protective effects due to production of antimicrobial compounds including bacteriocins [4, 52]. Moreover, the elucidation of the draft genome of *E. faecium* M3K31 has confirmed the existence in this strain of the EntHF biosynthetic cluster, the enterocin P structural and immunity genes, and a gene encoding the putative antimicrobial peptide SRCAM 602 [53]. Accordingly, *E. faecium* M3K31 producing EntHF and free of defined virulence markers, would be also considered a safe probiotic for further biotechnological applications including animal nutrition.

## Conclusions

This study has determined that enterococci is the largest LAB group with bacteriocinogenic activity, isolated from griffon vulture feces. However, the absence of LAB with antagonistic activity against Gram-negative bacteria suggest that bacteria, other than LAB, would be investigated for their activity against this bacterial group. Most enterococci encoded multiple bacteriocins although is production seems to be regulated by still unknown and deficiently evaluated mechanisms. Moreover, *E. faecium* M3K31 has been identified as producer of EntHF, a bacteriocin with remarkable antimicrobial activity against most evaluated *Listeria* spp. and elevated interest as a natural food preservative. *E. faecium* M3K31, absent of defined virulent markers, would be also considered a safe probiotic for use in animal nutrition.

## Additional file

Additional file 1: Figure S1. Comparative eBURST analysis against whole MLST from *E. faecalis* (A) and *E. faecium* (B). The indicated ST sequences belong to hospital-adapted clonal complexes (CC). In Figure 1A, ST6 belongs to clonal complex 2 (CC2) and ST9 to CC9. In Figure 1B, the ST17, ST18 and ST78 belong to CC17, CC18 and CC78, respectively. **Table S1.** Primers and PCR conditions for bacteriocin amplification used in this study. References for Table S1. (DOC 655 kb)

## Abbreviations

ADT: Agar diffusion test
BLAST: Basic local alignment search tool
CC: Clonal complex
CLSI: Clinical and laboratory standards institute
EFSA: European food safety authority
ERIC-PCR: Enterobacterial repetitive intergenic consensus sequences- Polymerase chain reaction
KAA: Kanamycin aesculin azide
LAB: Lactic acid bacteria
MALDI TOF/TOF MS: MALDI TOF tandem mass spectrometry
MALDI-TOF MS: Matrix-assisted laser desorption-ionization time-of-flight mass spectrometry
MIC: Minimum inhibitory concentration
MLST: Multilocus sequence typing
MPA: Microtiter plate assay
MRS: de Man, Rogosa and Sharpe
NCBI: National center for biotechnology information
PCR: Polymerase chain reaction
RP-FPLC: Reverse phase-Fast protein liquid chromatography SB, Slanetz and Bartley
SOAT: Stab-on-agar test
TSA: Trypticase soy agar
UPGM: Unweighed-pair group method
