# Supplementary material for: Evaluation of bacteriocinogenic activity, safety traits and biotechnological potential of fecal lactic acid bacteria (LAB), isolated from Griffon Vultures (Gyps fulvus subsp. fulvus)
Source: BMC Microbiol. 2016 Sep 29;16:228. doi: 10.1186/s12866-016-0840-2 (PMC5041338; doi:10.1186/s12866-016-0840-2)
Supplement: Additional file 1: Figure S1. — Comparative eBURST analysis against whole MLST from E. faecalis (A) and E. faecium (B). The indicated ST sequences belong to hospital-adapted clonal complexes (CC). In Figure 1A, ST6 belongs to clonal complex 2 (CC2) and ST9 to CC9. In Figure 1B, the ST17, ST18 and ST78 belong to CC17, CC18 and CC78, respectively. Table S1. Primers and PCR conditions for bacteriocin amplification used in this study. References for Table S1. (DOC 655 kb) [file 12866_2016_840_MOESM1_ESM.doc]

**Legend to Figures (Suplementary)**

**Figure 1S.** Comparative eBURST analysis against whole MLST from *E. faecalis* (A) and *E. faecium* (B). The indicated ST sequences belong to hospital-adapted clonal complexes (CC). In Figure 1A, ST6 belongs to clonal complex 2 (CC2) and ST9 to CC9. In Figure 1B, the ST17, ST18 and ST78 belong to CC17, CC18 and CC78, respectively.


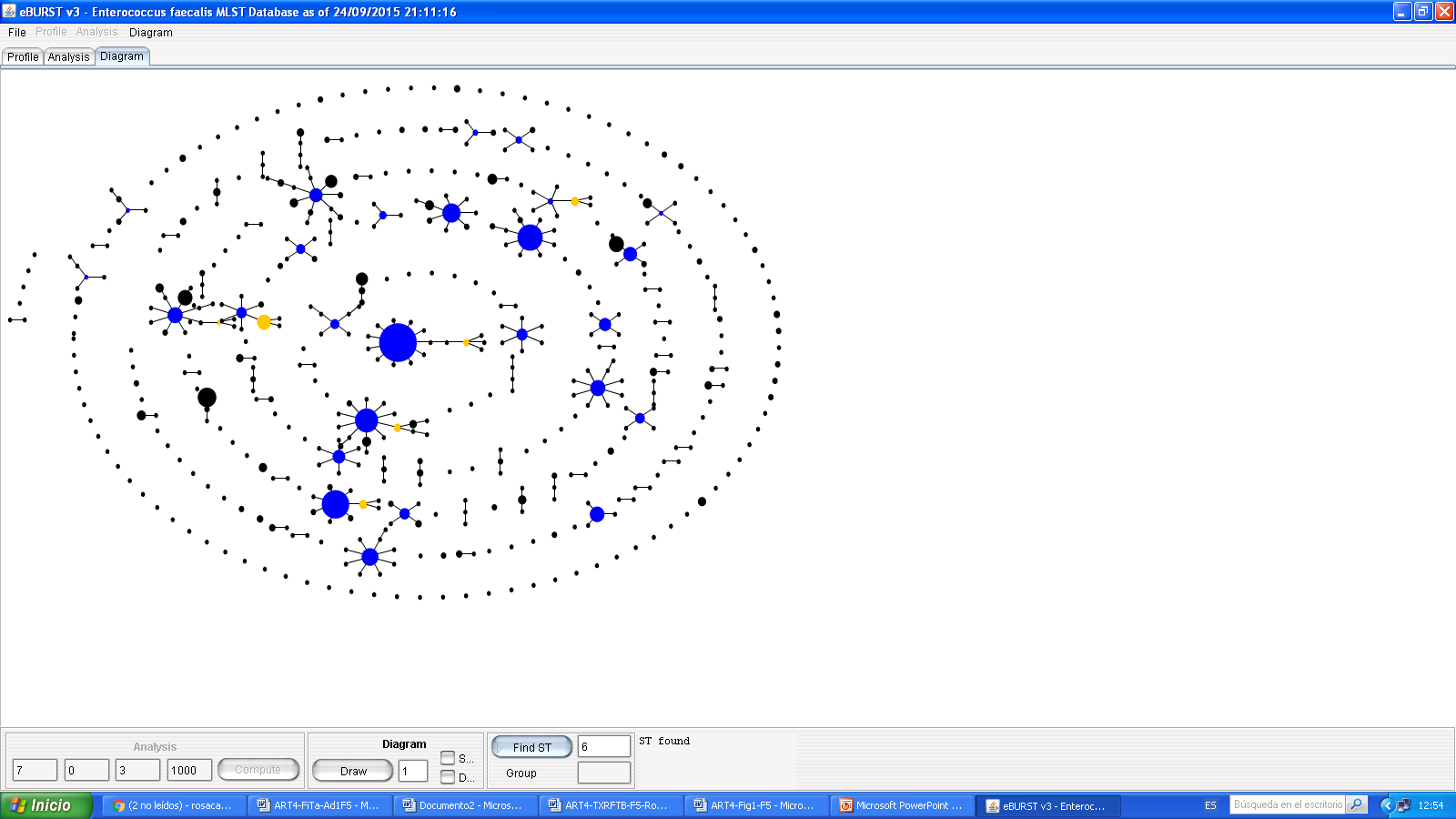


**ST6**

**ST167**

**ST68**

**ST9**

A


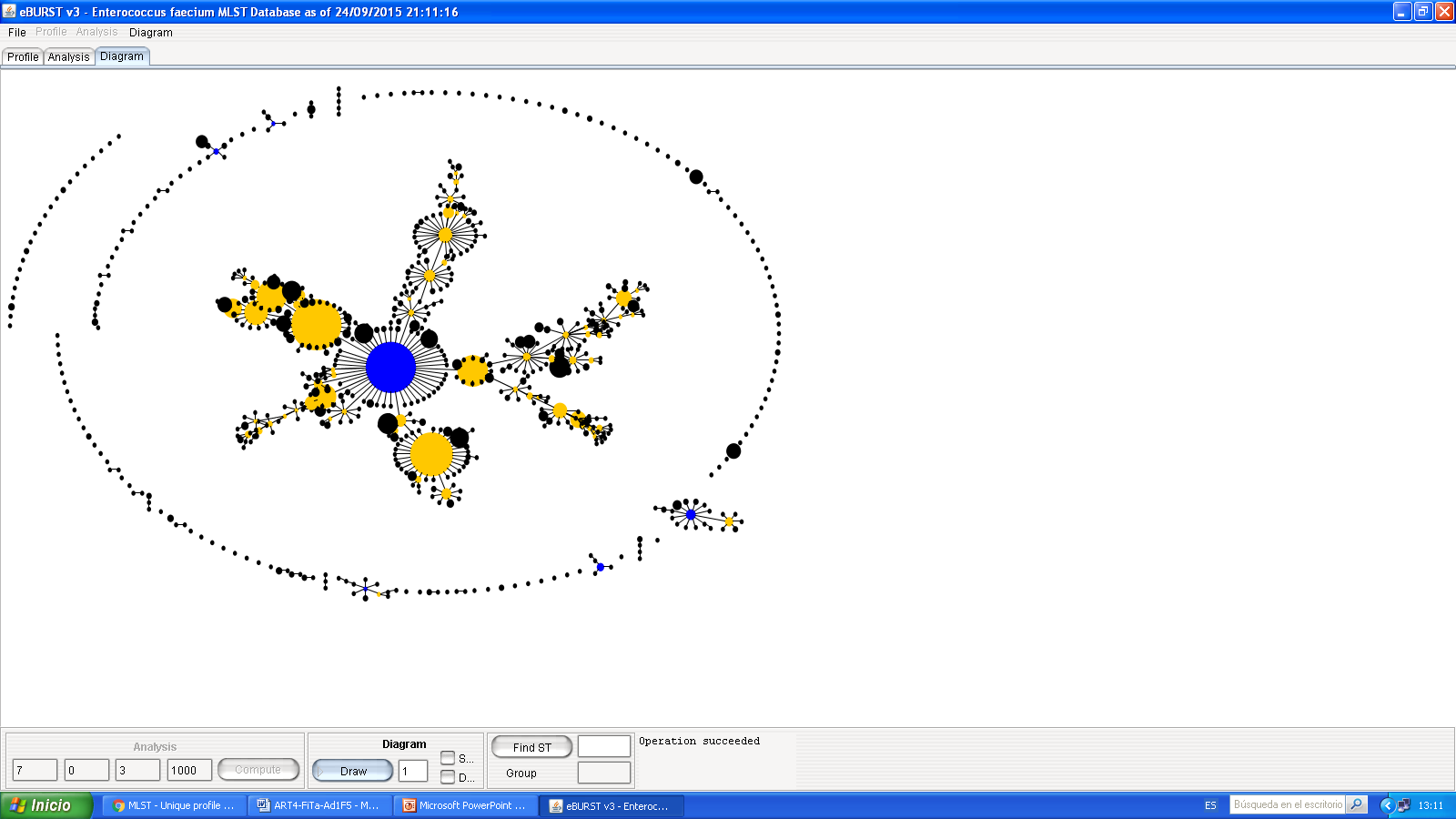


**ST22**

**ST78**

**ST18**

**ST76**

B

| **Table 1S.** Primers and PCR conditions for bacteriocin amplification used in this study | | | | | | |
| --- | --- | --- | --- | --- | --- | --- |
| Bacteriocin  (Reference)/Target gene | Primer | Nucleotide sequence (5’-3’) | Annealing  T (ºC) | PCR positive control  (Reference) | PCR product length (bp) | Primer reference |
| Avicin A [1]/ *avi*F | AVI-F  AVI-R | GTTGTTGGTGGTACCTACTATGG  GTAGACATGAACTCACCACC | 53ºC | Not available | 191 | This work |
| Bacteriocin 31 [2]/ *bac*31 | SB31-F  SB31-R | TTGTGGCATTATTGGGATTG  GCCATGTTGTACCCAACCA | 56°C | Not available | 166 | This work |
| Bacteriocin MC4-1 [3]/ *bac*A | MC4-F  MC4-R | CGGGATTGGTTTTACAGCAT  ATGCTGTAAAACCAATCCCG | 57ºC | Not available | 135 | This work |
| Brevicin 925A [4]/ *breB* | BREV-F  BREV-R | CAGTTGATGAATTAGCATTGATTG  AGTGCATGCCGTGTAAGTTG | 56°C | Not available | 192 | This work |
| Enterocin 96 [5]/ *ent*96 | S96-F  S96-R | ATTAGAAAATGGTGTAGTAAATGCTG  TCATTACAACCAGCTGGATCAC | 56ºC | Not available | 183 | This work |
| Enterocin 1071 [6]/ *ent*1071 | S171-F  S1071-R | TAGGTCCAGCTGCTTATTGG  CAGGTCCTCCACCAGTAATTG | 56ºC | *E. faecalis* FAIR-E309 [6] | 199 | This work |
| Enterocin A [7]/ *ent*A | EnterA-F  EnterA-R | ATGAAACATTTAAAAATTTTGTCTATTAAAG  TTAGCACTTCCCTGGAATTGCTCC | 59ºC | *E. faecium* T136 [8] | 197 | [9] |
| Enterocin AS-48 [10]/  *ent*AS-48 | SAS48-F  SAS48-R | TTTTGGGGTTAGCCTTGTTTA  TGCTGCAGCGAGTAAAGAAA | 56ºC | *E. faecalis* INIA-4 [11] | 191 | This work |
| Enterocin B [8]/ *ent*B | EntB3  EntB5 | AGACCTAACAACTTATCTAAAG  GTTGCATTTAGAGTATACATTTGC | 50ºC | *E. faecium* T136 [8] | 126 | [8] |
| Enterocin IT [12]/ *ent*IT | SIT-F  SIT-R | TGGTAGCTAGTTTGTGTTTGTTTAGC  GCAGTCCAACTCATAAGGTAACA | 56ºC | Not available | 213 | This work |
| Enterocin JS  (EF502034)/ *ent* JS | EntJSA-F  EntJSA-R | ATGGGAGCAATTGCAAAATTAGTAGC TCAATGTCTTTTTAACCATTTTTCAATTTGATC | 58ºC | *E. faecalis* DBH18 [13] | 310 | This work |
| Enterocin SE-K4 [14]/ *ent*SE | SEK4-FW  SEK4-RV | GCCACGTATTACGGAAATGGTGTC  TTATCTTCCACCTATACCACCTAACAC | 53ºC | *E. faecalis* FAIR-E309 [6] | 146 | [6] |
| Enterocin L50 [15]/ *ent*L50 | EntL50-R1  EntL50-R2 | ATGGGAGCAATCGCAAAATTAGTAGC  TTAATGTCTTTTTAGCCATTTTTCAAT | 65°C | *E. faecium* L50 [15] | 286 | [18] |
| Enterocin P [16]/ *ent*P | EntP1  EntP2 | ATGAGAAAAAAATTATTTAGTTTAGCTCTTATTGG  TTAATGTCCCATACCTGCCAAACCAG | 64ºC | *E. faecium* P13 [16] | 216 | [16] |
| Enterocin Q [17]/ *ent*Q | EntQ-R1  EntQ-R2: | ATGAATTTTCTTAAAAATGGTATCGCAAAATG  TTAACAAGAAATTTTTTCCCATGGCAAG | 57ºC | *E. faecium* L50 [17] | 105 | [18] |
| Enterococcin V583 [19]/ *ef*1097 | EF1097-F3  EF1097-R3 | GGCGATGGCATTACTAATGACATTAGG CTTAGCCCACATTGAACTGCCCATAAAGC | 65ºC | *E. faecalis* DBC5 [13] | 408 | [9] |
| Enterocin X [20]/ *ent*XA-XB | SXAB- F  SXAB-R | TGAAACAAATTATCGGTGGTTC  CTACGTCCACCATTCCAACC | 56ºC | Not available | 322 | This work |
| Enterolysin A [21]/ *enlA* | SENLA-F  SENLA-R | GGGTTTGACTTTGGTTCTGC  TTTTGGTCCTGGATTTGGAG | 56ºC | *E. faecalis* DBH9 [13] | 378 | This work |
| Hiracin JM79 [13]/ *hirJ*M79 | SHIR-F  SHIR-R | AGGAATATTAGGAACTTGTCTAGCTG  GGACCATGATTAACCCAACC | 56ºC | *E. hirae* DCH5 [13] | 171 | This work |
| Mundticin L [22]/ *mun*L | SML-F  SML-R | TGGGAAATACTACGGTAATGG  ACCAGCTGCTCCACCAGTAG | 56ºC | Not available | 124 | This work |
| Pediocin PA-1 [23*]/ pedA-1* | PA1-F  PA1-R | GAAATGGCCAATATCATTGGTG  CTAGCATTTATGATTACCTTGATGTC | 58ºC | *P. acidilactici* 347 [24] | 158 | This work |

**References for Table S1**

1. Birri DJ, Brede DA, Forberg T, Holo H, Nes IF. Molecular and genetic characterization of a novel bacteriocin locus in *Enterococcus avium* isolates from infants. Appl. Environ. Microbiol. 2010;76:483–92.

2. Tomita H, Fujimoto S, Tanimoto K, Ike Y. Cloning and genetic organization of the bacteriocin 31 determinant encoded on the *Enterococcus faecalis* pheromone-responsive conjugative plasmid pYI17. J. Bacteriol. 1996;178:3585–93.

3. Sedgley CM, Clewell DB, Flannagan SE. Plasmid pAMS1-encoded, bacteriocin-related “siblicide” in *Enterococcus faecalis*. J. Bacteriol. 2009;191:3183–8.

4. Wada T, Noda M, Kashiwabara F, Jeon HJ, Shirakawa A, Yabu H, et al. Characterization of four plasmids harboured in a *Lactobacillus brevis* strain encoding a novel bacteriocin, brevicin 925A, and construction of a shuttle vector for lactic acid bacteria and *Escherichia coli*. Microbiology 2009;155:1726–37.

5. Izquierdo E, Wagner C, Marchioni E, Aoude-Werner D, Ennahar S. Enterocin 96, a novel Class II bacteriocin produced by *Enterococcus faecalis* WHE 96, isolated from munster cheese. Appl. Environ. Microbiol. 2009;75:4273–6.

6. Franz CMAP, Grube A, Herrmann A, Abriouel H, Stärke J, Lombardi A, et al. Biochemical and genetic characterization of the two-peptide bacteriocin enterocin 1071 produced by *Enterococcus faecalis* FAIR-E 309. Appl. Environ. Microbiol. 2002;68:2550–4.

7. Aymerich T, Holo H, Håvarstein LS, Hugas M, Garriga M, Nes IF. Biochemical and genetic characterization of enterocin A from *Enterococcus faecium*, a new antilisterial bacteriocin in the pediocin family of bacteriocins. Appl. Environ. Microbiol. 1996;62:1676–82.

8. Casaus P, Nilsen T, Cintas LM, Nes IF, Hernández PE, Holo H. Enterocin B, a new bacteriocin from *Enterococcus faecium* T136 which can act synergistically with enterocin A. Microbiology 1997;143:2287–94.

9. Brandão A, Almeida T, Muñoz-Atienza E, Torres C, Igrejas G, Hernández PE, et al. Antimicrobial activity and occurrence of bacteriocin structural genes in *Enterococcus* spp. of human and animal origin isolated in Portugal. Arch. Microbiol. 2010;192:927–36.

10. Gálvez A, Maqueda M, Valdivia E, Quesada A, Montoya E. Characterization and partial purification of a broad spectrum antibiotic AS-48 produced by *Streptococcus faecalis*. Can. J. Microbiol. 1986;32:765–71.

11. Joosten HM, Nunez M, Devreese B, Van Beeumen J, Marugg JD. Purification and characterization of enterocin 4, a bacteriocin produced by *Enterococcus faecalis* INIA 4. Appl. Environ. Microbiol. 1996;62:4220–3.

12. Izquierdo E, Cai Y, Marchioni E, Ennahar S. Genetic identification of the bacteriocins produced by *Enterococcus faecium* IT62 and evidence that bacteriocin 32 is identical to enterocin IT. Antimicrob. Agents Chemother. 2009;53:1907–11.

13. Sánchez J, Basanta A, Gómez-Sala B, Herranz C, Cintas LM, Hernández PE. Antimicrobial and safety aspects, and biotechnological potential of bacteriocinogenic enterococci isolated from mallard ducks (*Anas platyrhynchos*). Int. J. Food Microbiol. 2007;117:295–305.

14. Eguchi T, Kaminaka K, Shima J, Kawamoto S, Mori K, Choi SH, et al. Isolation and characterization of enterocin SE-K4 produced by thermophilic enterococci, *Enterococcus faecalis* K-4. Biosci. Biotechnol. Biochem. 2001;65:247–53.

15. Cintas LM, Casaus P, Holo H, Hernandez PE, Nes IF, Håvarstein LS. Enterocins L50A and L50B, two novel bacteriocins from *Enterococcus faecium* L50, are related to staphylococcal hemolysins. J. Bacteriol. 1998;180:1988–94.

16. Cintas LM, Casaus P, Håvarstein LS, Hernández PE, Nes IF. Biochemical and genetic characterization of enterocin P, a novel sec-dependent bacteriocin from Enterococcus faecium P13 with a broad antimicrobial spectrum. Appl. Environ. Microbiol. 1997;63:4321–30.

17. Cintas LM, Casaus P, Herranz C, Håvarstein LS, Holo H, Hernández PE, et al. Biochemical and genetic evidence that *Enterococcus faecium* L50 produces enterocins L50A and L50B, the sec-dependent enterocin P, and a novel bacteriocin secreted without an N-terminal extension termed enterocin Q. J. Bacteriol. 2000;182:6806–14.

18. Citti, R. (2005). Aislamiento e identificación de bacterias lácticas bacteriocinogénicas de leches y quesos de búfala de Venezuela: actividad antimicrobiana y caracterización bioquímica y genética de sus bacteriocinas. PhD. Thesis. Universidad Complutense de Madrid. Madrid, Spain.

19. Swe PM, Heng NCK, Ting Y-T, Baird HJ, Carne A, Tauch A, et al. *ef1097* and *ypkK* encode enterococcin V583 and corynicin JK, members of a new family of antimicrobial proteins (bacteriocins) with modular structure from Gram-positive bacteria. Microbiology 2007;153:3218–27.

20. Hu C-B, Malaphan W, Zendo T, Nakayama J, Sonomoto K. Enterocin X, a novel two-peptide bacteriocin from *Enterococcus faecium* KU-B5, has an antibacterial spectrum entirely different from those of its component peptides. Appl. Environ. Microbiol. 2010;76:4542–5.

21. Nilsen T, Nes IF, Holo H. Enterolysin A, a cell wall-degrading bacteriocin from *Enterococcus faecalis* LMG 2333. Appl. Environ. Microbiol. 2003;69:2975–84.

22. Feng G, Guron GKP, Churey JJ, Worobo RW. Characterization of mundticin L, a class IIa anti-Listeria bacteriocin from *Enterococcus mundtii* CUGF08. Appl. Environ. Microbiol. 2009;75:5708–13.

23. Henderson JT, Chopko AL, van Wassenaar PD. Purification and primary structure of pediocin PA-1 produced by *Pediococcus acidilactici* PAC-1.0. Arch. Biochem. Biophys. 1992;295:5–12.

24. Martínez JM, Martínez MI, Suárez AM, Herranz C, Casaus P, Cintas LM, et al. Generation of polyclonal antibodies of predetermined specificity against pediocin PA-1. Appl. Environ. Microbiol. 1998;64:4536–45.
